# Design and Implementation of an Ultra-Low-Power Hazardous Gas Monitoring System

**DOI:** 10.3390/s25082458

**Published:** 2025-04-14

**Authors:** Hongyu Liu, Yuchen Wang, Jiankang Yu, Shuqing Wang, Huijuan Chen

**Affiliations:** 1 School of Safety Engineering, Huludao Campus, Liaoning Technical University, Huludao 125100, China; lhy991009@gmail.com (H.L.); shuqing618@gmail.com (S.W.); chenhj30114@163.com (H.C.); 2 School of Computer Science and Technology, Harbin Institute of Technology, Weihai 264209, China; pcsword@foxmail.com

**Keywords:** internet of things, ultra-low power consumption, hazardous gas monitoring, sensor systems

## Abstract

In order to effectively monitor harmful gas leakage, this paper presents the design of an ultra-low-power IoT-based harmful gas monitoring system. The system is equipped with a custom-designed, low-power microcontroller motherboard, carefully selected low-power sensors, and high-efficiency, low-power communication modules. In addition, the system optimizes data acquisition and processing algorithms to segment gases of different concentrations. While ensuring real-time data acquisition and transmission, it achieves extremely low power consumption. By controlling the concentration of harmful gases and current for sensor performance testing, the experiment has shown that when the concentration of carbon monoxide reaches 500 ppm and methane reaches 2000 ppm, the system will trigger an alarm and upload relevant information; the sensor can detect and respond to the harmful gases within 60 s; and the system’s operating current fluctuation range remains within 0.5 mA, with an average power consumption much lower than that of other devices.

## 1. Introduction

With the development of industrialization and urbanization, environmental safety issues have become increasingly important, especially the leakage of hazardous gases, which poses a serious threat to human health and public safety [1]. In industrial production, the operation of urban infrastructure, energy extraction, transportation, and other scenarios, real-time monitoring and early warning of hazardous gases have become necessary measures to ensure safe production and public health. In recent years, the advancement of Internet of Things (IoT) technology has led to the widespread application of various sensor-based hazardous gas monitoring systems [2,3,4,5,6]. These systems continuously monitor the concentration of harmful gases such as methane and carbon monoxide, enabling the timely detection of potential dangers and the prevention of disasters [7,8,9].

Currently, various monitoring technology solutions have been proposed and applied both domestically and internationally. For example, some systems adopt solutions based on wireless sensor networks, using low-power Bluetooth or ZigBee wireless communication technologies for data transmission, thus achieving low-cost, low-power transmission [10,11,12,13]. Other systems utilize cellular networks for data transmission, which offer advantages such as wide coverage and stable signals [14,15,16]. However, in the practical application of gas monitoring systems, sensors typically need to operate at high frequency or continuously to ensure the timeliness and accuracy of monitoring data. Such high-frequency sampling and data transmission often result in high power consumption and short battery life [17,18,19,20], particularly in harsh environments like Alxa and Xinjiang, where the maintenance of equipment and battery replacement become challenging tasks. For applications that require long-term unattended monitoring, such as urban underground gas leakage detection in pipelines and remote oil and gas pipeline leakage monitoring, wireless sensor networks can reduce the power consumption of data transmission but have limited coverage that cannot meet the needs of remote areas [21,22,23]. Although cellular networks [24] provide stable signals and extensive coverage, their high power consumption limits their application in long-term unattended scenarios. While these solutions have addressed wireless transmission issues to some extent, they still fall short in meeting the energy consumption demands of long-term monitoring and cannot simultaneously balance accuracy and power consumption.

To address this issue, low-power design has become an important direction for optimizing gas monitoring systems. This paper proposes an innovative ultra-low-power hazardous gas monitoring system that combines advanced hardware design with optimized power management strategies to progressively optimize the power consumption of the facilities. The aim is to fill the gap in existing technologies regarding low power consumption and long-term monitoring, meeting the specific needs of municipal gas systems and oil and gas facilities in remote areas for hazardous gas monitoring (according to the requirements of the municipal manhole system in Alxa, the sensor must trigger an alarm when the methane concentration reaches 2000 ppm and the carbon monoxide concentration reaches 500 ppm), thereby providing technical support for ensuring industrial safety and public health.

## 2. Materials and Methods

**Figure 1:**
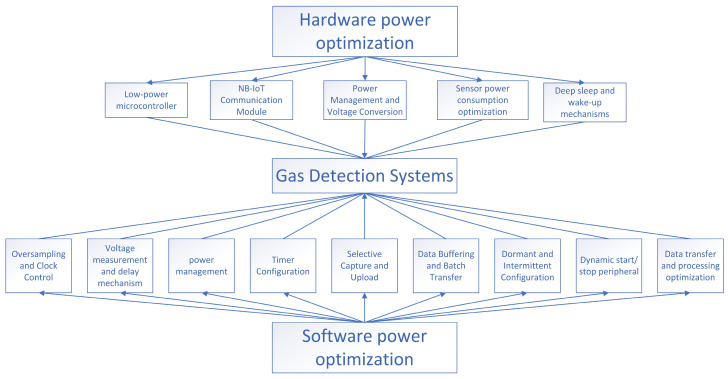
Power consumption optimization solution.

To address the challenge of low power consumption, this paper proposes optimizing the system’s power consumption incrementally from both hardware and software perspectives, thereby achieving an overall reduction in system power consumption. The solution is illustrated in Figure 1.

In terms of software optimization, unlike the dynamic memory allocation and release methods used by standard sensors, we have adopted a more efficient memory management strategy to avoid memory leaks and unnecessary memory allocations. This device employs memory pool and object pool technologies to reduce dynamic memory allocation, thus enhancing performance and lowering power consumption. Simultaneously, real-time power consumption monitoring is conducted to allow for immediate adjustments and optimization of energy consumption across various modules. Through algorithm optimization, unnecessary function calls have been reduced, and runtime overhead has been minimized as much as possible, thereby lowering CPU power consumption. Dynamic Voltage Frequency Scaling (DVFS), a technology used to switch the CPU core frequency based on load requirements, is applied to dynamically adjust parameters based on load to reduce energy consumption. Efficient communication protocols are chosen to optimize data transmission efficiency, enhancing overall performance.

In terms of hardware design, the system adopts ARM Cortex-M series microcontrollers and electrochemical sensors with low static and dynamic power consumption. In addition, we have introduced an integrated and efficient Power Management Unit (PMU), which can dynamically adjust the power state according to system requirements, keeping the device in sleep or power-off mode whenever possible to reduce overall system power consumption. In terms of circuit design, appropriate current-limiting components and buck converters are used to prevent current wastage, while also simplifying the circuit layout and reducing the number of series and parallel connections between components to enhance overall energy efficiency.

### 2.1. Software Power Consumption Optimization

#### 2.1.1. Sampling and Clock Control

By configuring the clock source and prescaler to reduce the sampling rate, and enabling oversampling to average multiple sampling points, measurement accuracy can be improved. Once sampling begins, the system triggers a conversion, reads multiple sample values upon completion, and calculates their average. Methane is sampled every 30 s using a rolling average method: the average concentration of the last three minutes is calculated and stored every minute. Once 240 concentration values have been accumulated, the maximum value among these is calculated and saved. Carbon monoxide is also sampled every 30 s, but the maximum value is extracted every 10 min for statistical analysis and upload. After sampling is complete, the system enters a low-power mode or reduces the sampling frequency to save energy. The conversion from analog to digital signal follows Formula (1):

(1) $${ADC}_{result}=\frac{V_{input}}{V_{ref}}\times \left(2^{resolution}-1\right)$$

where ${ADC}_{result}$ is the sampled value obtained from the ADC pin, $V_{input}$ is the voltage input to the ADC, $V_{ref}$ is the reference voltage of the ADC, and resolution refers to the resolution (bit width) of the ADC.

#### 2.1.2. Voltage Measurement and Delay Mechanism

In the voltage measurement and delay mechanism, the voltage measurement is triggered by initiating sampling, followed by calling a delay function to ensure that the system does not perform other tasks while waiting for the sampling conversion to complete. During this time, the system enters a low-power mode to reduce CPU activity. Once the delay ends, the sampling conversion results are read, and the obtained voltage values are further processed or applied. The sampling conversion is represented by Formula (2):

(2) $$V_{channel}=\frac{{ADC}_{sample}\times V_{ref}}{2^{n}-1}$$

where $V_{channel}$ is the actual voltage on the channel, ${ADC}_{sample}$ is the sampled value obtained from the ADC, $V_{ref}$ is the reference voltage of the ADC, and n is the resolution of the ADC.

#### 2.1.3. Data Collection and Processing Methods

The methane sensor is a battery-operated metal oxide sensor developed by Figaro. From a practical application perspective, we primarily focus on the presence of CH4 and whether it has reached critical alarm thresholds. Therefore, for production and testing convenience, we only calibrate the sensor for three key concentrations: 0 ppm, 4000 ppm, and 10,000 ppm. Since we only need a rough reference value for other concentrations in between, we do not require exact concentration values. Therefore, for ease of calculation, we assume a linear relationship between the sensor’s output voltage and the actual methane concentration within two concentration ranges: from 0 ppm to 4000 ppm, and above 4000 ppm. When the methane voltage V is less than or equal to $V_{1}$, which is the voltage value calibrated by the sensor at 4000 ppm, the concentration is calculated using Formula (3):

(3) $$\left\{\begin{array}{c} P=K_{1}\times V+B_{1}\\ K_{1}=\frac{4000-0}{V1-V0}\\ B_{1}=4000-K1\times V1 \end{array}\right.$$

where P is the required methane concentration, V is the voltage value to be converted into concentration, $K_{1}$ is the slope, representing the change in concentration corresponding to a unit change in voltage, $B_{1}$ is the intercept, representing the concentration when the voltage is zero, $V_{0}$ is the voltage value calibrated at 0 ppm methane concentration, and $V_{1}$ is the voltage value calibrated by the sensor at 4000 ppm.

When the methane voltage *V* exceeds $V_{1}$, the concentration is calculated by Formula (4):

(4) $$\left\{\begin{array}{c} P=K_{2}\times V+B_{2}\\ K_{2}=\frac{10,000-4000\;}{V2-V1}\\ B_{2}=10,000-K2\times V2 \end{array}\right.$$

where $V_{2}$ is the voltage value calibrated at 10,000 ppm methane concentration.

The carbon monoxide sensor is also an electrochemical sensor developed by Figaro. We calculate the concentration by combining the measured voltage value from the sensor with the sensor’s resistance and calibration coefficients. The calculation method and parameters for the CO sensor are provided by the sensor manufacturer. The manufacturer has calibrated each sensor before shipping, including the calculation coefficient at a CO concentration of 500 ppm (after testing and using in actual scenarios, our circuit design has been confirmed to exhibit no zero current). Similar to the methane measurement requirements, we are primarily concerned with the presence of CO and whether it reaches 500 ppm; other CO values are only used as references. The concentration is calculated using Formula (5):

(5) $$P_{CO}=\frac{V_{CO}\times 1000^{2}}{R_{CO}\times {\;K}_{CO}}$$

where $P_{CO}$ is the concentration of carbon monoxide, $V_{CO}$ is the voltage value, $R_{CO}$ is the resistance value, and $K_{CO}$ is the coefficient used for calculating the carbon monoxide concentration, printed on the sensor by the manufacturer at the time of production.

When the sensor is operational, external interference may cause noise or transient abnormal values, and directly using raw data can easily affect the accuracy of measurement results. To address this, we decided to save multiple historical data points and reduce the impact of random noise by averaging, thus making the measurement data more stable and reliable. The value is calculated by Formula (6):

(6) $$Average=\frac{1}{n}\sum_{i=1}^{n}values\left[i\right]$$

where n is the size of the array, $values\left[i\right]$ is the *i*-th element of the array, and Average is the returned average value.

#### 2.1.4. Battery Voltage Calculation

To ensure battery power monitoring, this paper proposes periodic sampling of the battery voltage to determine whether to enter low-power mode or trigger a low battery warning. We assume that the actual battery voltage is $V_{battery}$, and the voltage measured after passing through a voltage divider circuit is $V_{measured}$. The actual voltage can be calculated using Formula (7):

(7) $$V_{battery}=V_{measured}\times \frac{R_{1}+R_{2}}{R_{2}}$$

where $R_{1}$ and $R_{2}$ are the two resistors in the voltage divider circuit.

### 2.2. Hardware Design Optimization

To meet the requirements of devices that operate over long periods and low bandwidth, this design incorporates a low-power wide-area communication module to ensure rapid entry into a low-power state after data transmission is complete, reducing battery consumption. Through tight integration with the microcontroller and optimized power management, the overall energy consumption of the system is effectively reduced. In terms of power management, the motherboard utilizes a multi-level voltage conversion circuit that employs voltage stabilizing diodes and filtering capacitors to ensure the stability of the voltage supply and enhance the system’s anti-interference capabilities, improving device stability in complex environments and reducing energy wastage. The sensor module employs a pulse heating mode to reduce the power consumption of continuous power supply and to increase response speed. In addition, the mainboard is equipped with power control pins that can cut off the power supply to sensors when real-time monitoring is not required, thereby reducing the operating time of system components and further lowering overall power consumption.

## 3. System Design

### 3.1. Overall Framework

**Figure 2:**
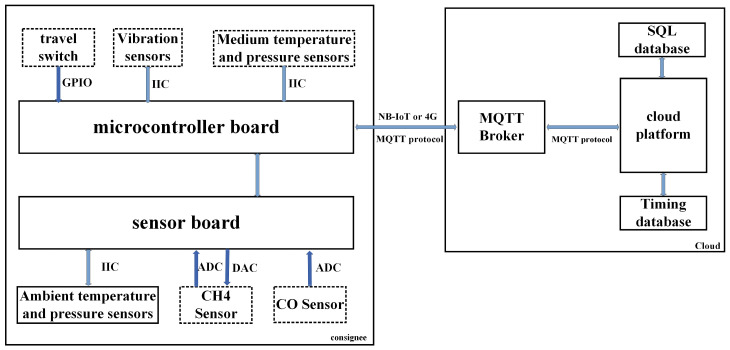
System architecture diagram.

In the design of this system, the overall architecture includes two main components: the edge device and the cloud, as shown in Figure 2. The edge device collects environmental data through various sensors, and these data are transmitted to the cloud’s MQTT Broker via NB-IoT or 4G networks. Finally, the data are stored in the cloud database using the MQTT protocol for further analysis and processing.

The edge device architecture consists of a microcontroller mainboard, a sensor board, and various sensor modules, forming the core data collection and processing unit of the system. The microcontroller mainboard acts as the central processing unit, interacting with different types of sensors through various interfaces such as I2C, GPIO, and ADC. It also uploads the collected sensor data to the cloud’s MQTT Broker using NB-IoT or 4G communication modules, enabling remote data transmission and monitoring. The sensor board integrates signals from various sensor modules and transmits them to the microcontroller mainboard for centralized processing. The integrated sensor modules include: limit switches for detecting the physical travel state of the device; vibration sensors for monitoring the device’s vibration; medium pressure (which refers to the pressure inside the gas pipeline) and temperature sensors; environmental pressure and temperature sensors for capturing external pressure and temperature data; a CH4 sensor for detecting methane concentration; and a CO sensor for monitoring carbon monoxide concentration in the environment. These sensor modules work together to provide real-time monitoring of the device’s operating status and surrounding environmental parameters, offering crucial data support for the system’s safety and stability.

In the cloud architecture, the cloud platform receives various sensor data from the edge device via protocols and stores the data according to type. Specifically, structured data are stored in an SQL database for easier querying and analysis, while time-series data related to the device are stored in a time-series database to support in-depth, time-based analysis and processing. This dual-database architecture not only enhances the flexibility of data management but also meets the storage and processing needs of different data types, thereby providing robust support for the overall performance and data analysis capabilities of the system.

Through the collaboration between the edge device and the cloud, this system provides real-time monitoring, data transmission, and cloud storage capabilities, effectively detecting the device’s travel status and various environmental parameters while reliably transmitting this data to the cloud for processing and storage. The cloud platform supports querying and analyzing historical data through a persistent storage mechanism, ensuring long-term monitoring and data traceability. Additionally, the system enables remote management and data analysis through the cloud platform, supporting applications in environmental monitoring and device status monitoring, particularly suitable for long-term, remote monitoring and data processing scenarios, with broad applicability and high reliability.

### 3.2. Hardware Design

#### 3.2.1. Mainboard Design

**Figure 3:**
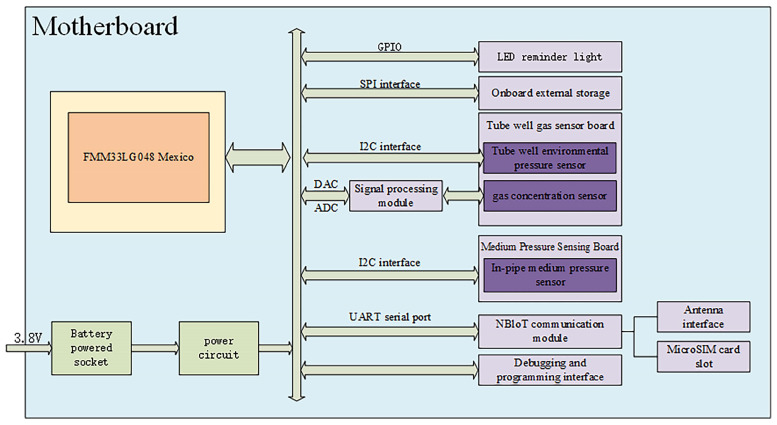
Motherboard block diagram.

In the design of the hazardous gas monitoring system, the low-power design of the motherboard, careful hardware selection, and optimization of power management strategies are key to ensuring the long-term stable operation of the system. The product features a robust metal casing and a protective coating on the PCB surface. All internal gaps are filled and sealed with silicone sealant, which not only prevents the minimal moisture in the gas pipelines (which remain dry and virtually moisture-free) from affecting the circuitry but also improves EMC sensitivity. Additionally, by optimizing the layout in the motherboard design, the length and crossing of signal lines have been effectively reduced, further enhancing EMC sensitivity. As shown in Figure 3, a modular architecture has been adopted, integrating multiple interfaces to meet the requirements of complex embedded applications.

The power supply utilizes a multi-level voltage conversion circuit, complemented by voltage stabilizing diodes and filters. This design not only ensures a stable voltage supply but also enhances electromagnetic compatibility and anti-interference capabilities. This design effectively reduces noise interference, ensuring that the motherboard remains stable under harsh conditions such as high temperature, high humidity, and strong electromagnetic interference. Additionally, by leveraging hardware sleep mode and on-demand power supply technology, the power consumption of the motherboard is further reduced, providing robust support for the system’s long-term, continuous operation.

#### 3.2.2. Communication Module Configuration

**Figure 4:**
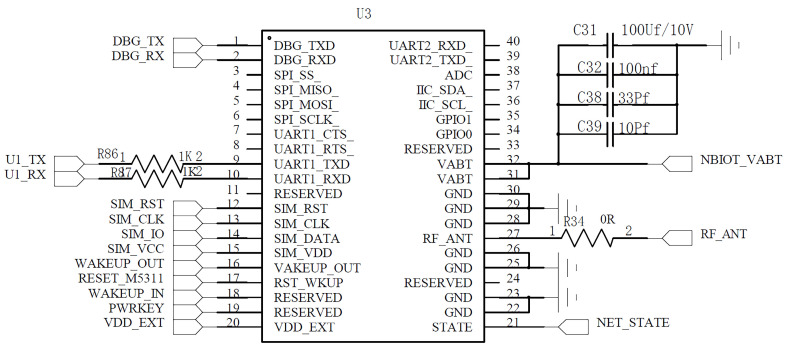
Circuit schematic diagram of NB-IoT communication module.

In this system, the communication module is closely integrated with the microcontroller, achieving further reduction in system power consumption through optimized interface design and power management strategies. The low-power characteristics of the communication module are evident not only in its microamp-level current consumption in standby mode, but also in its efficient communication protocol, which allows it to quickly enter a low-power state after data transmission is completed, thereby maximizing battery life. The circuit diagram of the module is shown in Figure 4.

#### 3.2.3. Sensor Module Design

**Figure 5:**
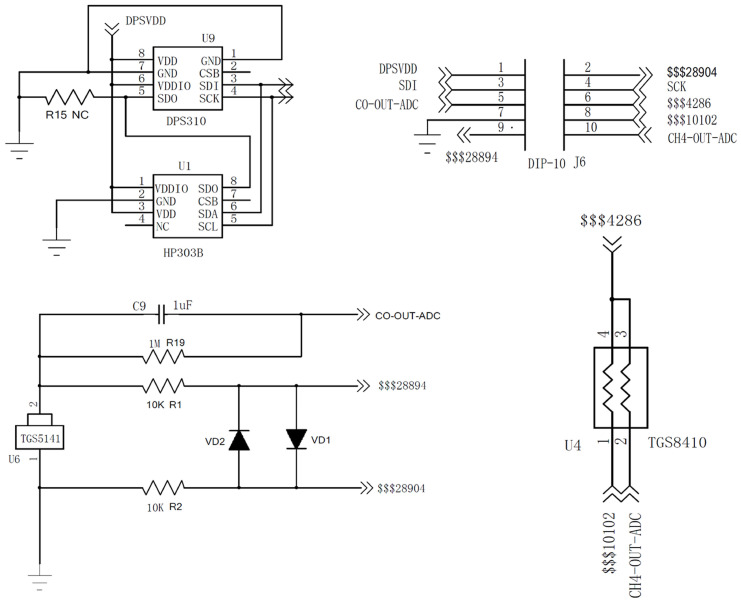
Module schematic diagram.

To achieve optimal performance of the sensors, the system incorporates a dedicated sensor module. The schematic diagram of the module is shown in Figure 5. The sensor board is connected to the motherboard through a 10-pin socket, adopting a modular design that facilitates installation and maintenance. It is equipped with an efficient preheating circuit and signal output circuit to ensure the sensor quickly reaches its operating temperature upon startup and provides stable output signals during measurements.

In the methane sensor design, a pulse heating mode is employed, in which periodic voltage pulses control the heating temperature of the detection element. This design not only significantly reduces power consumption but also improves the sensor’s response speed and extends its lifespan. For the carbon monoxide sensor, a high-precision signal amplification circuit is designed in combination with its output signal range and high-sensitivity characteristics, ensuring the accuracy and stability of the measurement data.

The sensor module also integrates anti-interference and data filtering technologies, such as hardware filtering circuits and software-based moving average algorithms, which effectively suppress environmental noise and short-term signal fluctuations. Through the careful selection and configuration of the methane and carbon monoxide sensors, combined with an optimized sensor board design, the system achieves high-precision gas detection while further reducing power consumption, laying a solid foundation for long-term and stable gas monitoring.

### 3.3. Data Collection and Processing

#### 3.3.1. Data Collection Process

**Figure 6:**
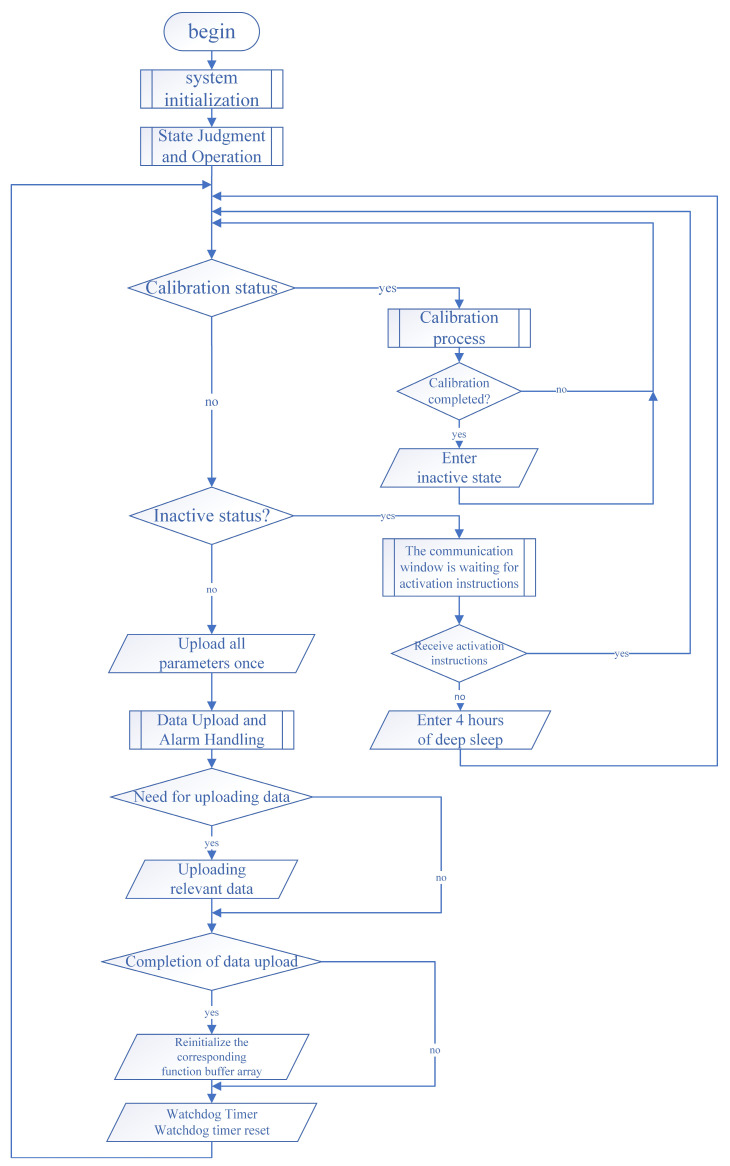
Data acquisition flowchart.

The data collection process outlined in this article consists of several steps to ensure the completeness and accuracy of the data, as detailed in Figure 6.

Upon system startup, the device first initializes various functions, including reading and writing parameters stored in Flash memory, which pertain to system configuration and the initial state of the sensors. Then, the system proceeds based on whether the device has been activated. If the device is not activated, it enters a communication window mode, waiting to receive activation commands. Meanwhile, the NB-IoT state machine checks the network connection status and performs reconnection or restart operations as necessary.

Once the device is activated, the system initiates vibration sensor data processing and manhole cover detection procedures to gather necessary environmental data. It smooths the data from the pressure sensor, calculates absolute values, and completes related calculations for energy density and gauge pressure. After processing the data, the device evaluates whether to upload periodic data and high-frequency pressure data based on preset conditions. If these conditions are met, the data are uploaded and resent in the event of a transmission failure to ensure successful data upload.

Furthermore, the device monitors environmental concentrations of methane and carbon monoxide. If the concentrations exceed predefined alarm thresholds, the system immediately uploads the data and switches to a low-energy deep sleep mode to reduce energy consumption and ensure safety. Finally, the device periodically undertakes high-frequency data collection and phased data processing. After completing a set of data collections, the system reinitializes the function buffer to prepare for the next round of data collection. If a sensor reading error occurs during collection, the device implements corresponding error-handling measures to ensure data accuracy and overall stability.

In the aforementioned process, this system employs techniques such as deep sleep, retry mechanisms, and periodic collection and processing to ensure that it operates only when necessary, significantly reducing the system’s power consumption.

#### 3.3.2. Calibration Process

**Figure 7:**
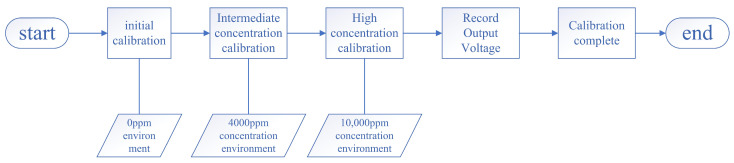
Multi-point intercalibration flow chart.

To ensure the measurement accuracy of the methane sensor under different concentration environments, the system is designed with a strict calibration process. This calibration process is divided into three stages, and the specific procedure is shown in Figure 7.

The intercalibration environment is a sealed chamber that can be filled with methane, in which a high-precision desktop concentration detector provides accurate reference values. The sensor is first placed in a methane-free environment (i.e., 0 ppm concentration) to begin the initial intercalibration stage. This stage lasts for at least 2.5 min. During this stage, the device’s indicator light alternates flashing every 5 s as a visual prompt. The device reads the sensor’s output voltage during the later part of this stage and processes the data, using the result as the reference voltage corresponding to 0 ppm. At the end of this stage, the indicator light turns off to indicate completion. Next, methane is gradually introduced into the intercalibration environment until the concentration reaches 4000 ppm. When a rise in concentration is detected (indicated by an 80 mV increase in voltage compared to the 0 ppm level), the device enters the medium concentration intercalibration stage. During this stage, the indicator light remains steadily on as a prompt. Once the concentration stabilizes, the device reads and processes the sensor’s output voltage over a period of time, using the result as the reference voltage corresponding to 4000 ppm. This stage ends with the indicator light turning off to indicate completion. Methane continues to be added to the intercalibration environment until the concentration reaches 10,000 ppm. When the device detects a further increase in concentration (a 50 mV increase in voltage compared to the 4000 ppm level), it enters the high concentration intercalibration stage. During this stage, the indicator light alternates between on and off every second as a prompt. Once the concentration stabilizes, the device reads and processes the sensor’s output voltage over a period of time, using the result as the reference voltage corresponding to 10,000 ppm. This stage ends with the indicator light turning off to indicate completion.

This multi-point intercalibration method enables the system to accurately calibrate the sensor response curve under different concentration environments, thereby improving measurement accuracy and stability. This specific approach involves recording the sensor’s output voltage at multiple concentration points, which serve as reference values for subsequent measurements. This method not only ensures real-time performance and accuracy of monitoring in low-power mode but also significantly reduces energy consumption, making the system more efficient and competitive than traditional engineering solutions. It is especially suitable for environments requiring long-term, unattended monitoring, where a low-power, high-precision monitoring system is essential.

#### 3.3.3. Concentration Calculation and Alarm Mechanism

The calculation of methane concentration is based on the calibrated voltage values stored in memory (0 ppm, 4000 ppm, and 10,000 ppm), fitting a function relationship that includes five parameters. The system measures the sensor voltage every 30 s to estimate the current methane concentration. Within the first 5 min after startup, the system calculates the average concentration over the past 3 min every minute and accumulates these data. After storing 240 concentration values, the system calculates and stores the maximum value, which is then uploaded as periodic data to the upper-level system.

Regarding the over-limit alarm mechanism, the system stores the latest 84 maximum values in a circular array, using the maximum value as the alarm threshold. When the methane concentration exceeds the threshold by 2000 ppm for five consecutive readings, the system triggers a “methane concentration over-limit alarm” and uploads the information. If the concentration exceeds 10,000 ppm, a higher-level alarm is triggered, and data collection is paused for 4 h. During the alarm period, if the concentration is below the alarm threshold for five consecutive readings, the system triggers an event to indicate the “methane concentration over-limit alarm cleared”.

For the calculation of carbon monoxide concentration, the system uses stored coefficients to implement it through fitting a single-parameter function relationship. The system collects data every 10 s and calculates the current concentration every 10 min, recording the maximum value for that period. Concentration data is uploaded every 4 h. When the carbon monoxide concentration reaches or exceeds 500 ppm, the system triggers an alarm and uploads the relevant information. For every additional 2000 ppm, the system updates the alarm information accordingly. When the concentration drops below 500 ppm, the system triggers an event signaling “carbon monoxide concentration alarm cleared”.

#### 3.3.4. Data Communication Module

**Figure 8:**
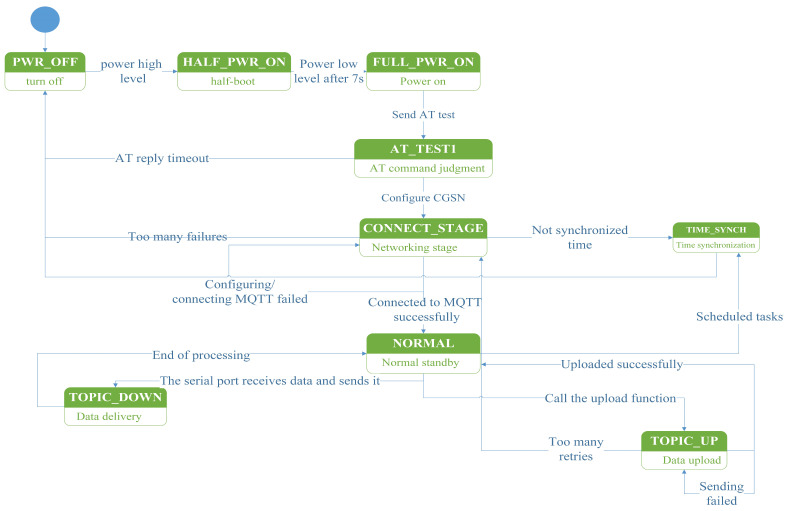
State machine diagram.

In Internet of Things (IoT) applications, efficient data upload and low-power operation of devices are crucial for ensuring the long-term stability and reliability of the system. The NB-IoT (Narrowband Internet of Things) module, designed specifically for low-power wide-area networks (LPWAN), effectively achieves low power consumption and efficient communication through its refined state machine control and intermittent data upload strategy, as illustrated in the state machine diagram in Figure 8.

The NB-IoT module utilizes a sophisticated state machine process in device operation management, covering the complete sequence from device startup and network connection to entering a normal standby state. The transitions between different states ensure precise control over each operational phase, especially during data uploads. The system employs a strict communication process in low-power conditions to achieve efficient and accurate data transmission.

The design of the communication protocol fully accounts for low power consumption requirements. This paper adopts the MQTT protocol and encodes the payload directly using HEX byte streams to reduce data traffic and communication time overhead. Additionally, a state machine module is used to centrally manage connection and data transmission. The system enters deep sleep mode when communication is not required and wakes only at key communication points, thereby significantly reducing power consumption. Before each communication, the module performs connection tests and parameter configuration checks via AT commands to ensure data is uploaded in the shortest possible time, avoiding prolonged resource occupancy that leads to additional energy consumption. Figure 8 details the complete process of the state machine, clearly marking each stage from system startup to the completion of data upload.

Additionally, the NB-IoT module employs an intermittent data upload mechanism that not only effectively reduces device power consumption but also ensures the accuracy and timeliness of data transmission. This low-power communication mode lays a solid technical foundation for the long-term stable operation of IoT devices. Experimental results show that this system reduces power consumption by half compared to traditional solutions, while ensuring data transmission efficiency. This significantly extends device battery life, reduces maintenance costs, and contributes significantly to the energy efficiency and environmental sustainability of IoT devices.

## 4. System Performance Analysis

### 4.1. Construction of the Experimental Platform

**Figure 9:**
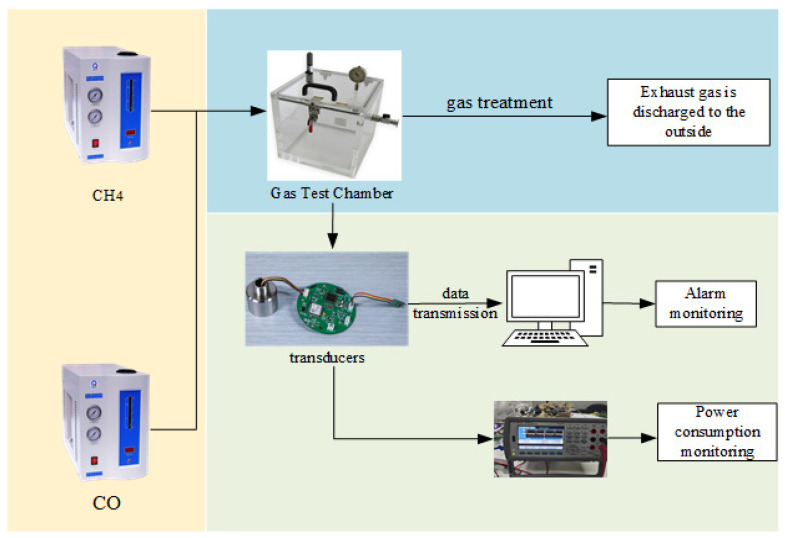
Experimental platforms.

To verify the performance of our system, we constructed a specialized experimental platform, as shown in Figure 9. This platform includes the following equipment: a gas generation device, a gas test chamber, hazardous gas sensors, a data monitoring terminal, and an oscilloscope.

The experimental environment simulated various scenarios found in actual applications, including changes in gas concentration and fluctuations in environmental temperature, to assess the performance of the system under different conditions. By comparing the time when gas exceeds standard limits in the gas reaction chamber with the time it takes for alarms to be uploaded to the cloud, we determined the response time of the sensors. In addition, we conducted an extensive survey of existing sensor devices on the market and determined that other low-power devices have response times of 60 s or less and average power consumption of approximately 30 mA. Subsequently, we used a power consumption monitoring instrument to monitor the working current of our sensor and compare its power performance against existing products.

### 4.2. Experimental Process and Results

**Figure 10:**
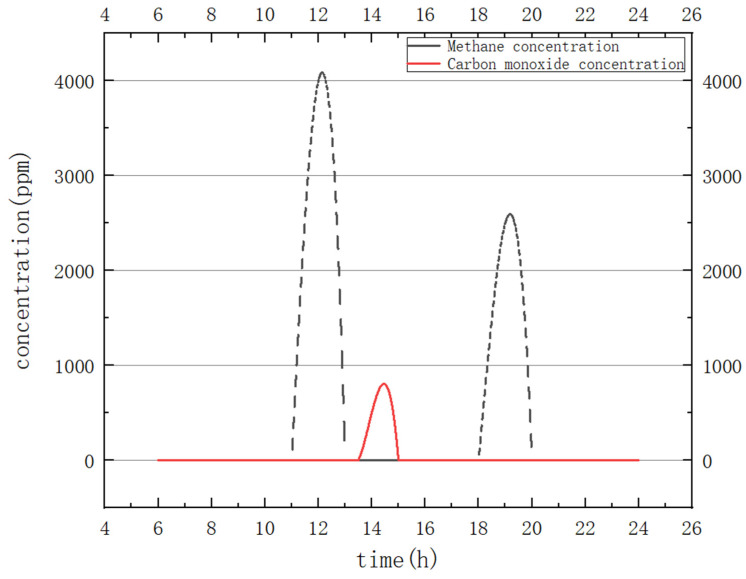
Gas concentration variation graph.

During the experimental process, we first tested the system’s response capability by artificially injecting gas into the test chamber to simulate a gas leak event. This ensured that the system could monitor changes in gas concentration in real time and issue immediate alarms when the concentration exceeded limits. The variations in methane and carbon monoxide concentrations within the gas test chamber are shown in Figure 10. The sensor’s feedback to these gas changes is presented in Table 1.

The experimental results demonstrate that when the gas concentration exceeds the predefined limits, the sensors capture and upload the data to the system within 60 s, and they accurately trigger an alarm for the exceeded gas levels. The alarm times for the sensors used in the power consumption comparison experiment ranged between 30 s and 120 s; therefore, the response time of our sensors meets the operational requirements.

**Figure 11:**
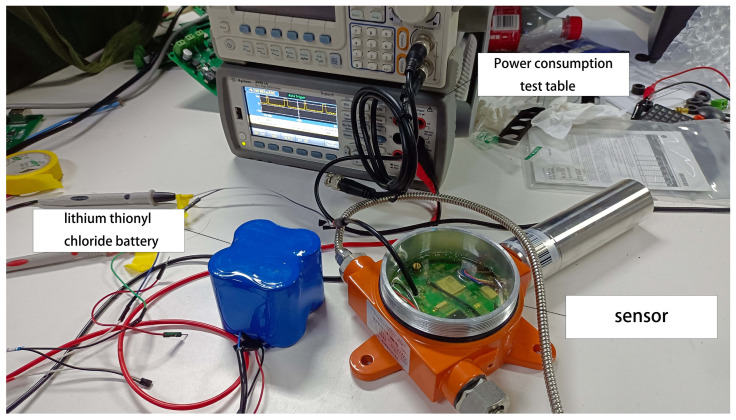
Current test environment setup.

**Figure 12:**
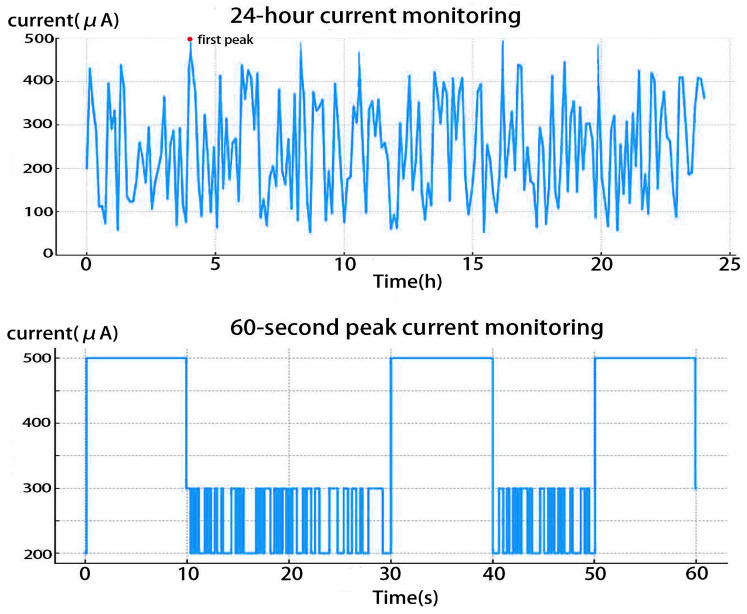
Running state power consumption test chart.

After verifying the sensor’s immediate and accurate gas detection and alarm capabilities, we conducted detailed tests on the system’s power consumption performance. In a classroom environment (as shown in Figure 11), the sensor was equipped with a lithium thionyl chloride battery. Lithium thionyl chloride batteries are primary batteries and do not require temperature compensation. We used four 3.6 V, 19 Ah batteries, totaling 72 Ah, which can theoretically provide power for five years. The choice of this battery type was based on the actual application scenario. The tests included measuring the sensor’s power consumption over a continuous 24 h operation period and capturing the peak power consumption generated by the first signal sent within 60 s. The results are shown in Figure 12.

After unit conversion, test results indicate that the power consumption of our system is only around 0.5 mA, far lower than other products on the market, and only one-sixtieth of the market average. Due to this low power consumption characteristic, compared to commonly available systems that require maintenance or battery replacement every few months or semi-annually, the battery life of our system can be extended to about two years. This significantly reduces the frequency of battery replacements and maintenance costs, demonstrating outstanding energy efficiency advantages.

## 5. Conclusions

This paper presents the design and development of an ultra-low-power sensor system for hazardous gas monitoring. It analyzes actual timestamp data and, based on this analysis, develops an effective statistical filtering method and piecewise linear regression technique to precisely estimate sensor sampling timestamps related to the system clock. This achieves a significant reduction in power consumption during the transmission of multi-parameter environmental data, demonstrating the potential to construct a low-power, high-accuracy, and near-real-time NB-IoT signal transmission system. The sensor stores segmented data for different concentrations of methane, issuing a danger alarm when levels exceed 2000 ppm, while the monitoring range for carbon monoxide is set at 500 ppm, with alarms triggered when this threshold is exceeded. Simulated experiments have shown that the process from sensor detection of gas exceeding limits to uploading data and issuing cloud alarms is completed within 60 s. Measurements show that the current fluctuation of our sensors remains around 0.5 mA, only one-sixtieth that of other sensors on the market, thereby enhancing the work efficiency of gas detection in remote areas.

## Figures and Tables

**Table 1:** Information feedback form.

Data Upload Time	Data Upload Content	Response Time
7:00	normal	
11:24	methane concentration exceeds limit alarm	32 s
12:57	methane concentration over-limit alarm is cleared	
14:00	carbon monoxide alarm	35 s
14:11	carbon monoxide alarm	27 s
14:52	carbon monoxide concentration alarm cleared	
18:42	methane concentration exceeds limit alarm	30 s
19:51	methane concentration over-limit alarm is cleared	
24:00	normal	

## Data Availability

If data support is needed, please contact pcsword@foxmail.com.
